# Prevalence of Congenital Malformations among Babies Delivered at a Tertiary Care Hospital

**DOI:** 10.31729/jnma.4985

**Published:** 2020-05

**Authors:** Sabina Shrestha, Anup Shrestha

**Affiliations:** 1 Department of Pediatrics, Kathmandu Medical College and Teaching Hospital, Kathmandu, Nepal

**Keywords:** *congenital malformations*, *live births*, *Nepal*, *prevalence*

## Abstract

**Introduction:**

Congenital malformations have emerged as a major cause of stillbirths and neonatal mortality. It is a common cause of morbidity and mortality not only in the newborn but also in childhood and beyond. The objective of this study was to find the prevalence of congenital malformation at birth.

**Methods:**

The descriptive cross-sectional study was conducted among 2456 live births in Kathmandu Medical College and Teaching Hospital from April 2017 to March 2018 after obtaining ethical approval from the institutional review committee (Ref no. 08052017). A convenient sampling method was applied. All the live-born babies delivered in this hospital during the study period were clinically examined for the presence of congenital anomalies. All malformations were classified according to the International Classification of Diseases-10 classification. The mothers of the newborns with congenital malformations were interviewed in a predesigned proforma. Statistical analysis was done using statistical package for the social sciences version 20.

**Results:**

Out of 2456 examined live births, congenital malformations were observed in 66 cases. The prevalence of congenital malformation was 66 (2.6%) at 95% confidence interval (4.19-1.98) of total live births. The genitourinary system was the most common system involved with congenital malformations being 16 (24.2%), followed by musculoskeletal system 14 (21.2%), and cardiovascular system 12 (18.2%).

**Conclusions:**

Congenital malformation plays a major role in the mortality and morbidity of neonates as well as children. The genitourinary system was the most common system involved.

## INTRODUCTION

A congenital malformation (CM) or birth defect, is defined as any abnormality, either structural or functional, present at birth, which may have been inherited genetically, acquired during gestation, or inflicted with parturition.^1^ The etiology of congenital malformation may be genetic (30-40%) or environmental (5-10%). The cause remains unidentifiable in about 50% of the cases (idiopathic).^2^

With the control of infectious diseases and nutritional insufficiency, genetic disorders are coming to the forefront. Congenital malformations are a common cause of morbidity and mortality not only in the newborn period but well into childhood and beyond. Therefore, congenital malformations need to be identified and intervened early to save lives and prevent sufferings.^3^

The objective of the study is to find the prevalence of congenital malformations occurring among institutional live births at a tertiary care hospital of Kathmandu.

## METHODS

A descriptive cross-sectional study was conducted in Kathmandu Medical College and Teaching Hospital during a period of one year from April 2017 to March 2018.

Ethical approval was obtained from the institutional review committee of Kathmandu Medical College before initiation of the study (Ref no. 08052017). All the live-born babies delivered in this hospital during the study period were included. Stillbirths were not included in this study. All the live births were clinically examined for the presence of gross congenital malformations. Suspected malformations were subjected to further investigations for final diagnosis, which included echocardiography, ultrasonography, and computerized tomography scan. Sixty-six babies were found to have some forms of congenital malformation. A convenient sampling method was applied.

Sample size was calculated by the formula,

n= Z^2^ × p × q / e^2^

= (1.96)^2^ × 0.5 × 0.5 / (0.02)^2^

= 2400

Where,

n= sample size
Z= 1.96 at 95% Confidence Interval
q= 1-p
e= margin of error, 2%

Hence, the calculated sample size was 2400 but a total of 2456 samples were included in the study.

The malformations were classified according to the International Classification of Diseases (ICD-10) classification. The newborns diagnosed with gross congenital malformations were managed as per protocol. Mothers of the newborns with congenital malformations were interviewed in a predesigned proforma after obtaining informed consent. The variables included maternal age, antenatal checkup, antenatal history of drug intake, consanguinity, birth weight of the newborn, and previous history of malformations. Statistical analysis was done using Statistical Package for the Social Sciences (SPSS) version 20.

## RESULTS

The prevalence of congenital malformation obtained was 66 (2.6%) at 95% confidence interval (4.19-1.98) of total live births. There were a total of 2480 deliveries during the study period. The total number of live births during the same period was 2456 (99.03%) and stillbirths were 24 (0.97%). The male to female ratio was 1.13. There were 131 (5.3%) preterm babies and 2349 (94.7%) were term babies. The frequency and sex distribution of the study population is shown (Table 1).

**Table 1:** Demographic profile: frequency and sex distribution.

	Total births n (%)	malformations n (%)
**Births**		
Total births	2480 (100)	66 (2.6)
Total live births	2456 (99.03)	66 (2.6)
Still births	24 (0.97)	Not included
**Sex**		
Males	1317 (53.10)	45 (3.4)
Females	1163 (46.89)	21 (1.8)

The maximum number of malformations was seen involving the genitourinary system 16 (24.2%). This was followed by a musculoskeletal system 14 (21.2%), and cardiovascular system 12 (18.2%). The distribution of congenital malformations in various systems is shown (Table 2).

**Table 2:** Distribution of congenital malformations in each system.

System involved	n (%)
Musculoskeletal system	14 (21.2)
Central nervous system	6 (9.1)
Cleft lip and cleft palate	6 (9.1)
Genitourinary system	16 (24.2)
Eye, ear, and face	6 (9.1)
Digestive system	3 (4.5)
Cardiovascular system	12 (18.2)
Syndromes	2 (3)
Multiple birth defects	1 (1.5)

Hypospadias 8 (12.1%) and atrial septal defect 8 (12.1%) were the most common malformations observed during the study, followed by polydactyly 7 (10.6%), congenital hydronephrosis 4 (6.1%), undescended testis 4 (6.1%), cleft lip and palate 3 (4.5%) and facial dysmorphism 3 (4.5%). The systems commonly involved and the subsequent congenital malformations in each system is shown (Table 3).

**Table 3:** Systems involved and the commonest malformations in each system.

System involved	Type of malformation	n (%)
Musculoskeletal	Congenital talipes	4 (6.1)
system	euinovarus	
	Polydactyly	7 (10.6)
	Oligodactyly	1 (1.5)
	Absent left hand	1 (1.5)
	Genu recurvatum	1 (1.5)
	Total	14
		(21.2)
Central nervous	Meningomyelocele	1 (1.5)
system	Spina bifida occulta	1 (1.5)
	Hydrocephalus	2 (3)
	Choroid plexus cyst	1 (1.5)
	Hypoplastic cerebellar vermis with dilated cisterna magna	1 (1.5)
	Total	6 (9.1)
Cleft lip and cleft	Cleft lip	2 (3)
palate	Cleft palate	1 (1.5)
	Cleft lip and palate	3 (4.5)
	Total	6 (9.1)
Genitourinary	Hypospadias	8 (12.1)
	Congenital hydronephrosis	4 (6.1)
	Undescended testis	4 (6.1)
	Total	16
		(24.2)
Eye, ear, and face	Preauricular sinus	1 (1.5)
	Preauricular tag, and sinus	1 (1.5)
	Facial dysmorphism	3 (4.5)
	Right anotia	1 (1.5)
	Total	6 (9.1)
Digestive system	Tracheoesophageal fistula	1 (1.5)
	Short segment Hirschsprung disease	1 (1.5)
	Congenital diaphragmatic hernia	1 (1.5)
	Total	3 (4.5)
Cardiovascular	Atrial septal defect	8 (12.1)
system	Ventricle septal defect	2 (3)
	Complex congenital heart disease	2 (3)
	Total	12
		(18.2)
Others: syndromes	Downs syndrome	1 (1.5)
	Stiff baby syndrome	1 (1.5)
	Total	2 (3)

## DISCUSSION

The prevalence of congenital malformations obtained in our study was 2.6%, which included both major and minor malformations detected at the time of birth. This rate was higher in comparison to the study conducted in the Western Regional Teaching Hospital, Pokhara (0.42%),^4^ Nepal, and Maternity Hospital (0.36%), Thapathali, Kathmandu.^5^ The rate was still higher than another study conducted in Patan hospital in the year 2014 (0.8%).^6^ However, the prevalence rate of congenital malformations obtained in our study is similar to the findings of Herbert A Obu, et al. (2.8%).^7^

The prevalence of congenital malformation varies to a great extent. In a study done in Northeast India, the prevalence of congenital malformation was obtained to be 1.2% of total live births.^8^ Rates as high as 2.5% in Egypt,^9^ 5.1% of live births in New York,^10^ and 5.1% in Wales^11^ have also been observed. The variations in the prevalence rates of congenital malformations in different studies may be due to differences in the study population, geographical variations, definition and classification of cases, diagnostic protocol and statistical calculation (the denominator).^12^

The study showed a higher incidence of congenital malformations among male babies (3.4%) than female babies (1.8%). Male preponderance has also been observed in other studies.^4^,^5^ Prematurity and low birth weight were found to be associated with a higher incidence of congenital malformations. However, these two factors may be the effect of congenital malformations, rather than the cause for it. A similar association was also observed by Ansari, et al. in their study.^6^

In the present study, the most common malformations were observed in the genitourinary system (24.2%), followed by the musculoskeletal system (21.2%) and cardiovascular system (18.2%). A similar study by Arya Singhe L, et al.^13^ showed that the most common congenital malformation involved the genitourinary system, followed by the musculoskeletal system. Likewise, a study by Malla BK^5^ showed that the most common system involved in congenital malformations were central nervous system, followed by musculoskeletal, gastrointestinal, genitourinary, and sense organ system. Congenital malformations of the central nervous system were also the commonest birth defect is a study done in North of Iran.^14^ Malformations of the musculoskeletal system were observed to be the commonest in a study conducted by Sharma I^4^ in Western Regional Hospital, Pokhara, Nepal, Ansari, et al.^6^ at Patan Hospital and Baruah^8^ in Northeast India.

As this was a descriptive cross-sectional study done in a tertiary care hospital, the prevalence calculated might not be projected to the general population, for which population-based studies are necessary. This study did not include abortions and stillbirths, because often the abnormalities are not obvious or visible externally. In those cases, a pathological autopsy is warranted, which was not feasible in our hospital setup and also in most of the cases, parental consent is not available for pathological autopsy.

## CONCLUSIONS

Congenital malformation plays a major role in the mortality and morbidity of neonates as well as children. It is important to identify the causative and risk factors of these problems and prevent them early. The genitourinary system was the commonest system involved.
